# Impact of contraction intensity and ankle joint angle on calf muscle fascicle length and pennation angle during isometric and dynamic contractions

**DOI:** 10.1038/s41598-024-75795-2

**Published:** 2024-10-22

**Authors:** Corinna Coenning, Volker Rieg, Tobias Siebert, Veit Wank

**Affiliations:** 1 Institute of Sports Science, Eberhard Karls University, Wilhelmstraße 124, 72074 Tubingen, Germany; 2 Department of Motion and Exercise Science, University of Stuttgart, Stuttgart, Germany; 3 Stuttgart Center of Simulation Science, University of Stuttgart, Stuttgart, Germany

**Keywords:** Muscle gearing, Ultrasound, AGR, Muscle architecture, Physiology, Musculoskeletal system, Muscle

## Abstract

During muscle contraction, not only are the fascicles shortening but also the pennation angle changes, which leads to a faster contraction of the muscle than of its fascicles. This phenomenon is called muscle gearing, and it has a direct influence on the force output of the muscle. There are few studies showing pennation angle changes during isometric and concentric contractions for different contraction intensities and muscle lengths. Therefore, the aim was to determine these influences over a wide range of contraction intensities and ankle joint angles for human triceps surae. Additionally, the influence of contraction intensity and ankle joint angle on muscle gearing was evaluated. Ten sport students performed concentric and isometric contractions with intensities between 0 and 90% of the maximum voluntary contraction and ankle joint angles from 50° to 120°. During these contractions, the m. gastrocnemius medialis and lateralis and the m. soleus were recorded via ultrasound imaging. A nonlinear relationship between fascicle length and pennation angle was discovered, which can be described with a quadratic fit for each of the muscles during isometric contraction. A nearly identical relationship was detected during dynamic contraction. The muscle gearing increased almost linearly with contraction intensity and ankle joint angle.

## Introduction

Human movement is the central object of research in sports biomechanics. In order to understand and study human movement, the quantification of muscle forces during movements is of primary importance. There are three different approaches for this quantification: measuring muscle forces during contraction by direct application of force measuring sensors to the tendon in vivo^1,2^, calculating muscle forces via geometric models of the joint using the inverse dynamics method to infer muscle forces from externally measured forces^3,4^, or calculating muscle forces within a movement simulated via specific muscle models^5–13^. Direct measurements are only possible with high experimental effort and within limits^1,2^. Furthermore, it is hardly ethically justifiable. Therefore, these two computational approaches have been favored in recent research. For all these approaches, it is important to consider the individual architectural parameters (e.g., muscle length, fascicle length, pennation angle) to infer the contraction dynamics of the muscle.

The architecture of the muscle must be reproduced as accurately as possible to be able to make reliable statements about its contraction dynamics. Because the fascicles in pennate muscles are not arranged along the force line of action of the muscle and therefore do not fully contribute to the effective force development, the pennation angle $\:{\alpha\:}_{F}$ is of great importance within the muscle architecture^8^. In classical muscle models, this angle is ignored or assumed to be constant^10,14^. However, numerous studies have shown that the fascicle angle changes as a function of muscle length (ankle joint angle position) and muscle contraction intensity^15–17^.

The exact relationship between muscle fascicle length $\:{l}_{F}$ and pennation angle $\:{\alpha\:}_{F}$ has only been reported in a few human studies. These few studies can be distinguished into the study of isometric and dynamic muscle contractions. For example, Heroux et al.^18^ investigated the relationship between $\:{l}_{F}$ and $\:{\alpha\:}_{F}$ of the m. gastrocnemius medialis (GM) and lateralis (GL) muscles at low contraction intensities (up to 25% maximum voluntary contraction (MVC)) during isometric contractions. Here, two ankle joint angles (90° and 120°), which directly influence the muscle length of the GM and GL, were considered. Narici et al.^19^ investigated the influence of the ankle joint angle on $\:{l}_{F}$ and $\:{\alpha\:}_{F}$ under passive conditions and the influence of different contraction intensities (0%, 20%, 40%, 60%, 80%, 100%) at 110° ankle joint angle. Furthermore, Kawakami et al.^20^ and Maganaris et al.^21^ investigated the influence of the ankle joint angle on the relationship between $\:{l}_{F}$ and $\:{\alpha\:}_{F}$ for the triceps surae (GM, GL, and m. soleus (SOL)). They found a nonlinear progression of the $\:{l}_{F}$ – $\:{\alpha\:}_{F}$ curve. In the study by Kawakami et al.^20^, only the conditions at rest and at maximal voluntary contraction (100% MVC) were investigated, whereas Maganaris et al.^21^ additionally investigated different contraction intensities for the 90° ankle joint angle. The triceps surae is mainly responsible for plantar flexion of the ankle joint^22^ and is essential for bipedal locomotion in everyday life and sports. However, very different contraction intensities occur in everyday life and especially in sports. Peak athletic performance is sometimes associated with maximal movement intensities (e.g., long jump) whereas walking is more likely to be associated with submaximal activity. In order to make a statement about the relationship between $\:{l}_{F}$ and $\:{\alpha\:}_{F}$ across the breadth of movement tasks, investigations over a wide intensity range (here 0 − 90% MVC) in combination with experiments over a broad spectrum of ankle joint angles (50°-120°) are necessary but lacking in the literature.

Studies investigating dynamic contraction behavior during walking^15,16,23–26^ have shown a quasi-isometric contraction of GL, GM and SOL muscle fascicles at onset followed by a concentric phase. Therefore, the $\:{l}_{F}$– $\:{\alpha\:}_{F}$ progression during a concentric contraction must also be considered to approximate a mapping of changes in muscle architecture. Lichtwark and Wilson^15^ and Lichtwark et al.^16^ examined the GM during concentric contraction and found a similar relationship between $\:{l}_{F}$ and $\:{\alpha\:}_{F}$ as in isometric contraction. However, investigations of the $\:{l}_{F}$ and $\:{\alpha\:}_{F}$ relationship during dynamic contraction for the GL and SOL are lacking in the literature.

Changing the pennation angle during contraction not only negatively affects the force development of the muscle but also shortens the muscle along its line of force action. This shortening allows the muscle fascicle to contract at slower velocities than the muscle belly (i.e. the muscle-tendon complex without the distal tendon). This effect is called “muscle gearing (G)” and is defined as the ratio of muscle velocity to muscle fascicle velocity according to Brainerd and Azizi^27^ and Eng et al.^28^. Muscle gearing > 1 has been shown in the GL of turkeys^29^ and the m. plantaris of frogs^30^. Additionally, muscle gearing was demonstrated in the GM of rats^31^. This effect was subsequently demonstrated in human GM^32^. Information on the occurrence of muscle gearing for the other muscles of the triceps surae, which is important for realistic model-based simulations of human movements, for example, is lacking.

In summary, the objectives of this study are (1) to demonstrate and model a dynamic $\:{l}_{F}$ – $\:{\alpha\:}_{F}$ progression of human GL, GM, and SOL as a function of contraction intensity (0 − 90% MVC) and ankle joint angle (50° − 120°) during isometric contractions, (2) to investigate $\:{l}_{F}$ – $\:{\alpha\:}_{F}$ progression during concentric contractions, and (3) to investigate muscle gearing as a function of muscle contraction intensity and ankle joint angles during isometric contraction.

## Methods

### Materials and methods

**Fig. 1:**
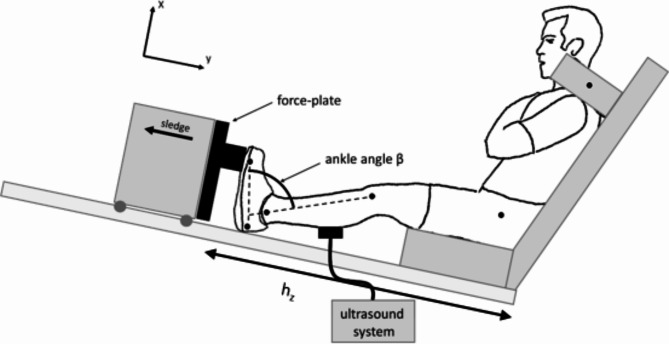
Experimental setup. Subjects performed plantarflexions in an instrumented leg extension machine. Force and ankle joint angle (β) were measured using force plates and video analysis. Optical markers (black points) were placed at lateral malleolus, lateral femoral condyle, first metatarsophalangeal joint, and calcaneus. Architectural data ($\:{\alpha\:}_{F}$, $\:{l}_{F}$) of the plantar flexors were measured using ultrasound. The sledge inclination was 11°.

Ten male sport students (27.1 ± 1.8 years, 79.2 ± 13.0 kg, and 1.81 ± 0.10 m) participated in the measurements to quantify the architectural parameters ($\:{l}_{F}$,$\:{\alpha\:}_{F}$) of the three main plantar flexors GL, GM, and SOL. The Ethics Committee at Eberhard Karls University in Tübingen approved the study protocol and all experiments were carried out in accordance to the guidelines and regulations of this committee (A2.5.4–254 _bi). All subjects were informed about the experimental procedure and the voluntary nature of participation before the start of measurements and provided written informed consent to participate in this experiment (A2.5.4–254 _bi). The measurements were performed in a seated position on an instrumented leg extension machine (Fig. 1), similar to the setup in Donath et al.^33^ and Ertelt and Blickhan^34^. The subjects were instructed to perform plantar flexion with their knees extended. Variation in the ankle joint angle (β) was possible by continuously adjustable sledge distance h_z_ (Fig. 1). The leg extension machine enabled isometric plantarflexion by fixation of the sledge as well as concentric plantarflexion with a free-moving sledge. Four optical markers were placed at predefined anatomical landmarks: lateral malleolus, lateral knee joint axis (located between the lateral condylus femoris and lateral condylus tibiae), first metatarsophalangeal joint, calcaneus. These markers were used to measure the ankle joint angle (angle between the axis from first metatarsophalangeal joint to calcaneus and the axis from lateral knee joint axis to lateral malleolus, Fig. 1) with a camera system (DR1 2048 × 1088-192-G2-8 von Photonfocus (Schweiz)) oriented perpendicular to the observation plane. Plantarflexion force was measured with a force plate attached to the sledge (Fig. 1), which was equipped with strain gauge sensors of type 85041 (Burster, Gernsbach) and S9 (HBM, Darmstadt) sensors to measure the reaction forces horizontally (y) and vertically (x), respectively. The analog signals from the force plate were processed via an amplifier (0.5 V, type AE101, HBM, Darmstadt) and a 16-bit AD converter (Data Translation 9032, 1000 Hz sampling rate) before being recorded via custom written analysis software.

Ultrasound recordings to determine the $\:{\alpha\:}_{F}$ and $\:{l}_{F}$ of GL, GM and SOL were performed using a Siemens ultrasound device (25 Hz, Sonoline G 50, Munich, Germany) and a linear probe (7.5 MHz L70) in brightness (B) mode. The ultrasound images and force data were synchronized via an external trigger, allowing accurate mapping of contraction intensity (% MVC) and muscle architecture. The cross-section area was measured using a spherical probe (5.0 MHz, C50).

### Experimental protocol

**Fig. 2:**
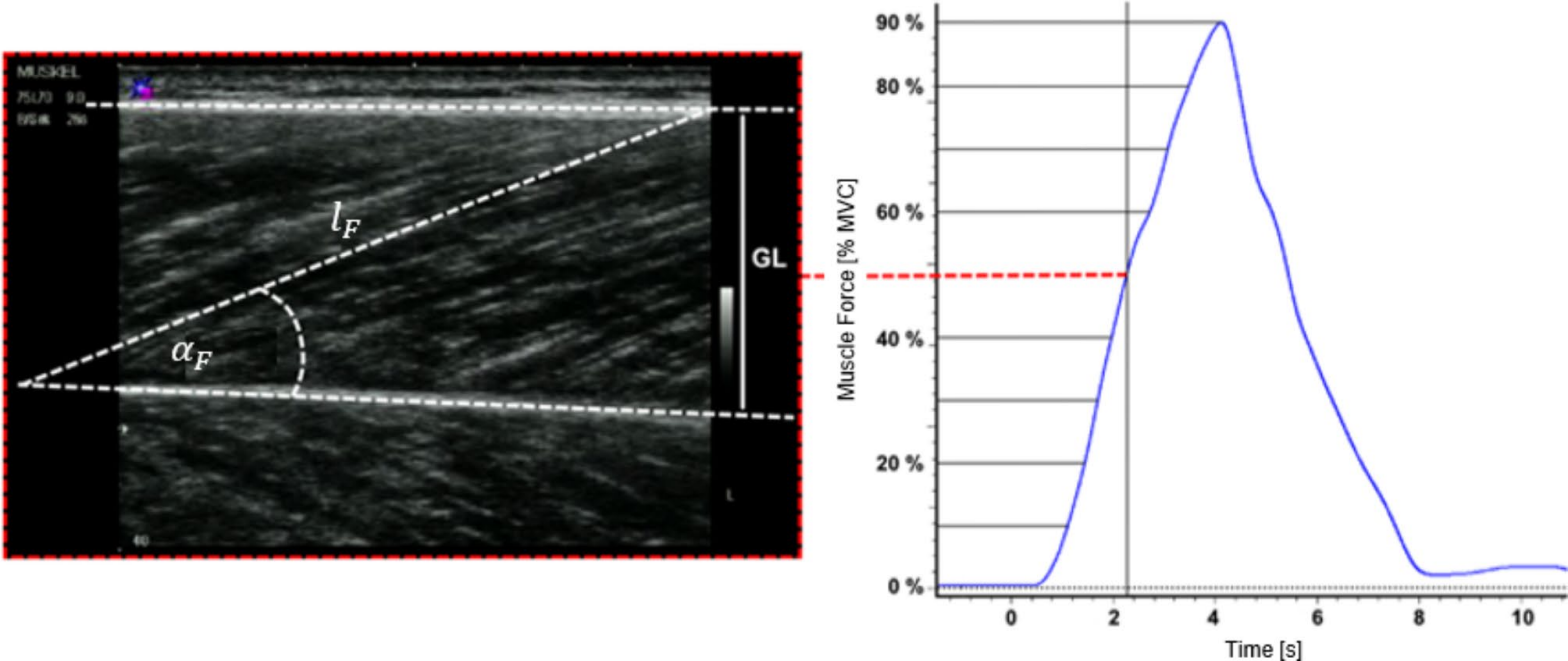
Determination of $\:{l}_{F}$ and $\:{\alpha\:}_{F}$ at 10 force increments (0–90% MVC in 10% steps). Subjects were instructed to increase force during an isometric plantarflexion task from zero to 90% MVC within 4 s (right). The left figure shows an ultrasound image of GL at 50% MVC. $\:{\alpha\:}_{F}$, $\:{l}_{F}$ and the proximal (upper) and distal (lower) aponeurosis are marked in white.

After a short familiarization phase, the subjects performed double-leg isometric plantar flexions at eight different ankle joint angles ranging from 50° to 120° (10° steps) in the leg extension machine (Fig. 1). In each case, three isometric voluntary plantar flexions were made per ankle joint angle:

(i) The first MVC was used to check for the correct ankle joint angle.
(ii) In a second MVC, changes in muscle architecture were measured via ultrasound.
(iii) In the third contraction, changes in muscle architecture were measured via ultrasound during a continuous increase in contraction intensity from rest (0% maximum isometric force, MVC) to 90% MVC within approximately 4 s (Fig. 2 right).

During the third contraction, a line at 90% MVC was superimposed on a computer monitor, and subjects received visual feedback of the current force generated to target this line. $\:{l}_{F}$ and $\:{\alpha\:}_{F}$ were recorded continuously by ultrasound at different contraction intensities, from rest to maximal voluntary contraction (MVC). Sufficient rest was maintained between each contraction to avoid fatigue effects. Ultrasound recordings at the three muscles were acquired separately for each muscle in turn.

Ultrasound measurements were recorded for GL and GM at the site of the largest anatomical cross-section area (CSA)^20,22,35^, and for SOL, the lateral part of the posterior portion was examined on the part with the biggest CSA, which GL or GM did not cover. Each muscle’s measurement position was marked on the skin surface at 90° ankle joint angle in the passive state. This was done to reproduce measurements at other ankle joint angle positions. Reproducing the same location is important to avoid measuring errors due to regional variations of the muscle architecture^36,37^. The ultrasound probe was attached perpendicular to the skin surface with as little pressure as possible so as not to influence contraction dynamics and muscle geometry by external pressure^38–41^. A water-soluble gel was applied to the transducer to avoid acoustic reflections. Ultrasound measurements continuously accompanied the entire contraction course, so the ultrasound probe only needed to be placed once per measurement. A Figure to illustrate the anatomy of the triceps surae is shown in the Supplementary Information as Supplementary Fig. S1.

When placing the ultrasound probe, care was taken to ensure that the individual muscle fascicles were clearly visible and, if possible, extended along their entire length from the superficial (proximal) to the deep-seated (distal) aponeurosis. This ensured that the ultrasound image depicted the plane of the fascicles and minimized measurement errors^39,42–45^.

Following the isometric contractions, each subject performed maximal concentric contractions against a sledge load of 125 kg. Due to the sledge inclination of 11° (Fig. 1), the load corresponded to approximately 30% of the body weight. The load was accelerated from rest beginning at an ankle joint angle of approximately 80°.

### Data analysis

Muscle fascicle lengths and pennation angles were recorded using custom-written software in Delphi. The force‒time curve was smoothed using a spline algorithm according to Reinsch^46^ before further evaluation. Muscle architecture parameters were quantified at 10 specific contraction intensities (10% increments from 0 to 90% MVC) at the different ankle joint angles.

In the ultrasound image, a representative fascicle was manually digitized with respect to its length and orientation, and the locations of both aponeuroses were determined. For each fascicle, a linear course without any curvature was approximated between the proximal and the distal aponeurosis^41^. $\:{l}_{F}$ was calculated from the intersection of the fascicles with the proximal and distal aponeuroses^47^. For very long fascicles that protruded from the image, the fascicle length was extrapolated assuming linearity of the fascicle and aponeuroses. Depending on the curvature of the fascicle, this approximation was accompanied by a maximum error of 2.4%^48^. The measured $\:{\alpha\:}_{F}$ was defined as the angle between the distal aponeurosis and the fascicle. Muscle Gearing was subsequently calculated as a result of the measured fascicle length and pennation angle.

1 $$\:G=\frac{{l}_{Muscle,2}-{l}_{Muscle,1}}{{l}_{F,2}-{l}_{F,1}}$$

2 $$\:G=\frac{\left(\text{cos}\left({\alpha\:}_{F,2}\right)*{l}_{F,2}\right)-\left(\text{cos}\left({\alpha\:}_{F,1}\right)*{l}_{F,1}\right)}{{l}_{F,2}-{l}_{F,1}}$$

The indices 1 and 2 indicate the timestep at which $\:{l}_{F}$ and $\:{\alpha\:}_{F}$ were taken. The 2 stands for either the next contraction intensity level or the next ankle joint angle, depending on the focus of the muscle gearing calculation^49^. Further analysis of the obtained data was performed using MATLAB software (R2017a, MathWorks).

### Statistics

One-way analysis of variance with repeated measurements ($\:\alpha\:\:$= 0.05) was used to check the intervention-related differences in the analyzed parameters ($\:{l}_{F}$, $\:{\alpha\:}_{F}$, and muscle gearing) in each group. In case of significance, a post hoc t-test for two dependent samples was performed ($\:\alpha\:\:$= 0.05). To investigate the agreement between the $\:{l}_{F}$-$\:{\alpha\:}_{F}$ relations of the concentric and isometric contractions, the coefficient of determination R^2^ was determined. R^2^ was calculated from the squared deviations between the polynomial fit to the isometric data (Fig. 6, dotted lines) and the data points from the concentric measurements (Fig. 6, coloured dots).

The data in all figures, tables and in the text are expressed as the mean and standard deviation (SD).

## Results

### Impact of ankle joint angle and contraction intensity on $\:{l}_{F}$ and $\:{\alpha\:}_{F}$ during isometric contractions

**Fig. 3:**
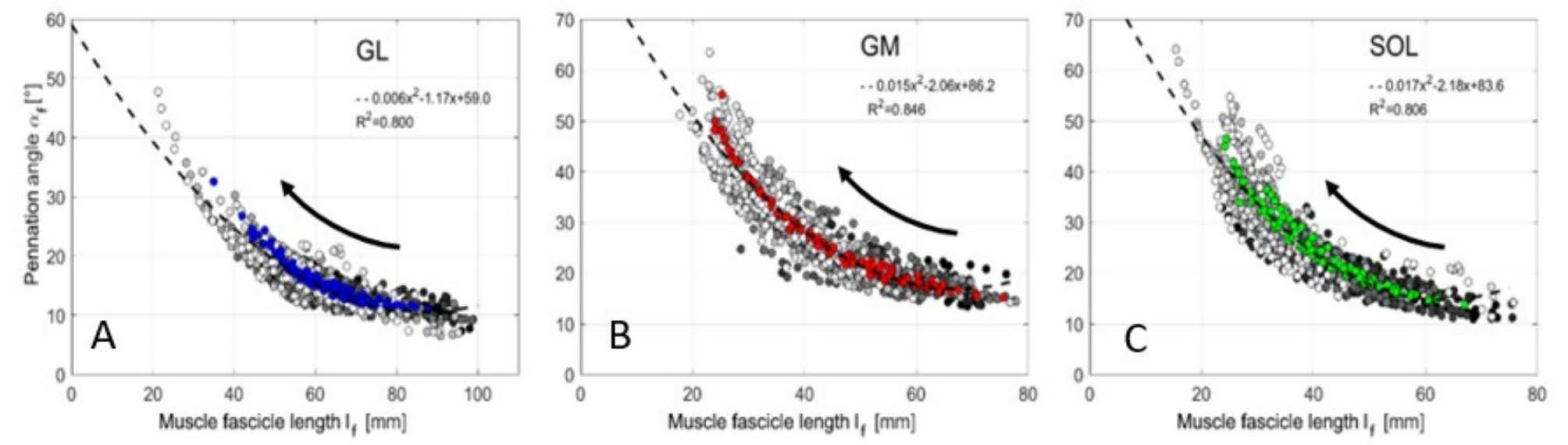
$\:{l}_{F}-{\alpha\:}_{F}$ relation of GL (**A**), GM (**B**) and SOL (**C**). The data of one gray scale correspond to an isometric experiment at one specific ankle angle. Consequently, there are 8 gray levels for 8 different ankle joint angles (50 to 120°, in 10° steps). $\:{l}_{F}$ –$\:{\alpha\:}_{F}$ data for contraction intensities from 0–90% MVC are displayed as dots in this shade of gray. Light gray and black correspond to 120° and 50° ankle joint angles, respectively. The isometric experiments begin in the passive state (0% MVC) with a high muscle fascicle length. With increasing contraction intensity (up to 90% MVC), there is a reduction in muscle fascicle length and an increase in pennation angle, which is visualized by the direction of the black arrow. The black broken line represents the polynomial fit (second degree) to all data points.

**Fig. 4:**
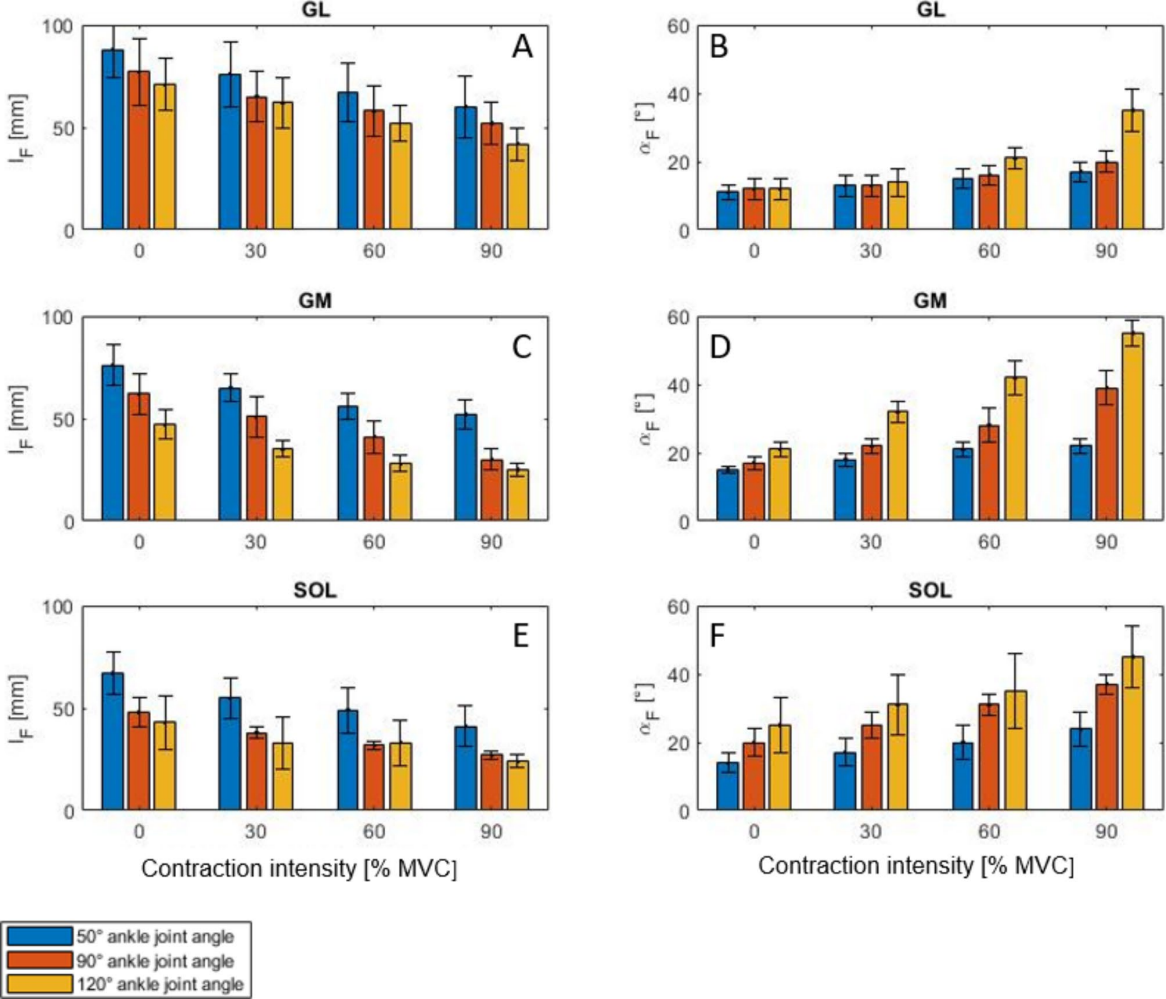
The impact of muscle contraction intensity on $\:{l}_{F}$ (**A**,** C**,** E**) and $\:{\alpha\:}_{F}$ (**B**,** D**,** F**) of GL (**A**,** B**), GM (**C**,** D**) and SOL (**E**,** F**). Each bar represents mean $\:{l}_{F}$ or $\:{\alpha\:}_{F}$. The standard deviation is represented in black. The different colored bars represent different ankle joint angles (blue: 50° ankle joint angle; red: 90° ankle joint angle; yellow: 120° ankle joint angle). Please note that for reasons of clarity, only four contraction intensities (0, 30, 60, 90%) and 3 ankle joint angles (50, 90, 120°) are shown. The complete data sets and statistics can be found in the supplement (Supplementary Table S1–S3).

Plotting all $\:{l}_{F}$-$\:{\alpha\:}_{F}$ combinations of GL for increasing muscle force (0-90% MVC) from isometric measurements revealed a nonlinear $\:{l}_{F}$-$\:{\alpha\:}_{F}$ relation (Fig. 3A). Measurements at specific ankle joint angles (50° to 120°) are illustrated by different shades of gray. For example, black points show isometric contraction at a 50° ankle joint angle. The black arrow in Fig. 3 illustrates the change in $\:{l}_{F}$-$\:{\alpha\:}_{F}$ combination with increasing muscle contraction intensity. At 50° ankle angle, the GL muscle fascicles decreased by 28 mm (*p* < 0.01, from 88 ± 14 mm to 60 ± 15 mm), and the GL pennation angle increased by 6° (*p* < 0.01, from 11 ± 2° to 17 ± 3°) with increasing contraction intensity (Fig. 4A, B, blue bars). In general, $\:{l}_{F}$ and $\:{\alpha\:}_{F}$ of GL increase significantly (*p* < 0.05, Supplementary Table S1) with muscle force during isometric contraction for each ankle joint angle.

With increasing ankle joint angle (from 50° to 120°) and thus decreasing muscle length, $\:{l}_{F}$*-*$\:{\alpha\:}_{F}$ relations of GL shift to shorter fascicle lengths and higher pennation angles. The shift to shorter fascicle lengths can be explained by a significant (*p* < 0.01) decrease in passive (0% MVC) starting fascicle length of 17 mm (Fig. 4A, from 88 ± 14 mm (blue bar, 0% MVC) to 71 ± 13 mm (yellow bar, 0% MVC) at 50° and 120° ankle joint angles, respectively). Interestingly, fascicle shortening during contraction (from 0 to 90% MVC) was independent of the ankle joint angle (*p* = 0.993; Δ$\:{l}_{F}$ = 28 ± 2 mm; Supplementary Table S1). The shift of GL $\:{l}_{F}$*-*$\:{\alpha\:}_{F}$ combinations to higher pennation angles can be explained by a significant (*p* < 0.01) increase in $\:{\varDelta\:\alpha\:}_{F}$ during contraction for increasing ankle joint angles. For example, with increasing contraction intensity from 0 to 90%, $\:{\varDelta\:\alpha\:}_{F}$ increases from 6° (at 50° ankle joint angle) to 15° (at 120° ankle joint angle) (Supplementary Table S1, right column).

GM and SOL showed overall similar behavior to GL (Fig. 3). However, muscle-specific differences were observed for $\:{\alpha\:}_{F}$ changes for GM and SOL, as well as for $\:{l}_{F}$ changes for SOL. The differences are described in the supplementary note. Details are presented in the supplementary information (Supplementary Tables S2 and S3).

The experimental $\:{l}_{F}$*-*$\:{\alpha\:}_{F}$ relations can be described by a polynomial fit (second degree) to all data points (R^2^ ≥ 0.8, Fig. 3).

3 $$\:{GL:\:\alpha\:}_{F}\left({l}_{F}\right)=\text{0.006}{l}_{F}^{2}-1.17{l}_{F}+\text{59.0}$$

4 $$\:{GM:\:\alpha\:}_{F}\left({l}_{F}\right)=\text{0.015}{l}_{F}^{2}-\text{2.06}{l}_{F}+\text{86.2}$$

5 $$\:{SOL:\:\alpha\:}_{F}\left({l}_{F}\right)=\text{0.017}{l}_{F}^{2}-\text{2.18}{l}_{F}+\text{83.6}$$

### Impact of ankle joint angle and contraction intensity on gearing (G) during isometric contractions

**Fig. 5:**
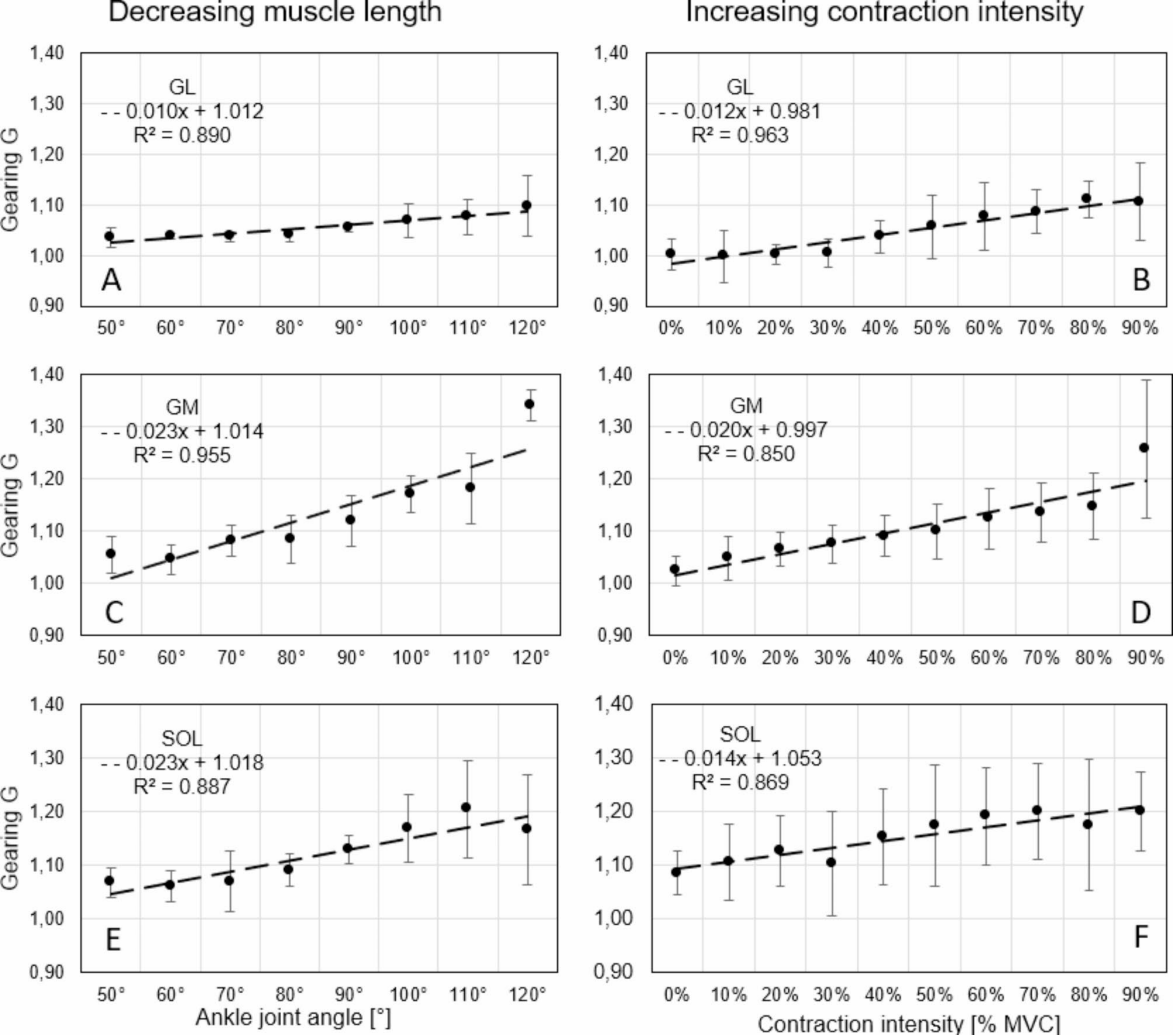
Impact of ankle joint angle (**A, C, E**) and muscle contraction intensity (**B, D, F**) (corresponding to muscle force in isometric contractions) for GL (**A**,** B**), GM (**C**,** D**), and SOL (**E**,** F**). Left column: each black point shows the mean gearing during contraction (0–90% MVC) for a single ankle joint angle. Right column: each black point shows the mean Gearing induced by changes in ankle joint angle from 50° to 120° for a given percentage of contraction intensity. Broken lines: linear fit of the data.

The impacts of the ankle joint angle and contraction intensity on G are shown in Fig. 5. Statistical analysis suggested that G increased with ankle joint angle and thus with decreasing muscle length (GL, *p* < 0.05; GM, *p* < 0.05; SOL, *p* < 0.05). A maximal G of 1.35 at 120° ankle joint angle was found for GM (Fig. 5C) compared to GL (1.1) and SOL (1.2) (Fig. 5A, E). Low Gearing values (approximately 1.05) were found for contraction intensities (from 0 to 90% MVC) at 50° ankle joint angles for all muscles (Fig. 5A, C, E). There was an almost linear relationship (R^2^ > 0.887) between G and the ankle joint angle.

Furthermore, we found that G increases with muscle force corresponding to contraction intensity in isometric contractions (GL, *p* < 0.05; GM, *p* < 0.05; SOL, *p* < 0.05). For GL, almost no gearing (1.01 ± 0.03) was found in the passive situation (0% MVC) when the ankle joint angle was changed from 50° to 120° (Fig. 5B), which can be explained by the lack of changes in passive $\:{\alpha\:}_{F}$ (Supplementary Table S1, left column). GM (Fig. 5D) and SOL (Fig. 5E) showed slightly greater G during passive changes in the ankle joint angle (GM 1.02 ± 0.03 and 1.07 ± 0.04), which can be explained by increasing $\:{\alpha\:}_{F}$ for increasing ankle joint angles (Supplementary Table S2 and S3, left columns). Similarly, there was an almost linear relationship (R^2^ > 0.850) between G and muscle force.

### $\:{l}_{F}$ and $\:{\alpha\:}_{F}$ during dynamic contractions

**Fig. 6:**
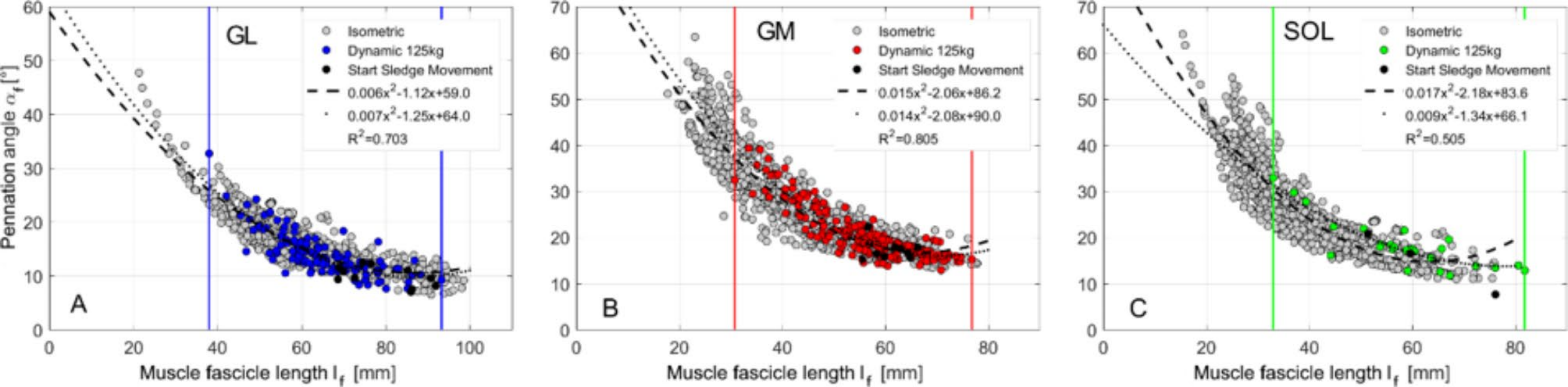
$\:{l}_{F}$ – $\:{\alpha\:}_{F}$ relation of concentric plantarflexions against sledge load of 125 kg of GL (**A**, blue circles), GM (**B**, red circles) and SOL (**C**, green circles). Gray circles are the $\:{l}_{F}$-$\:{\alpha\:}_{F}$ combinations from isometric contractions at all ankle joint angles (see Fig. 3). Dotted and broken lines correspond to polynomial fit (2. grade) of the data from isometric and dynamic experiments, respectively. Vertical colored lines represent the range of concentric plantarflexions.

Figure 6 shows the $\:{l}_{F}$-$\:{\alpha\:}_{F}$ relations from dynamic plantarflexions (colored circles, GL: blue, GM: red, SOL: green) against a sledge load of 125 kg. The contractions started at an approximately 80° ankle joint angle and were finished at approximately 120°. For all muscles (GL, GM, SOL), the $\:{l}_{F}$-$\:{\alpha\:}_{F}$ relations from dynamic measurements are within the data from isometric measurements (gray circles). Within the range of dynamic $\:{l}_{F}$-$\:{\alpha\:}_{F}$ combinations (vertical colored lines), the fitted curves (broken line: isometric data; dotted line: dynamic data) show good agreement for all three muscles. Due to the limited amount of dynamic SOL data (green circles), the shapes of the fitted $\:{l}_{F}$-$\:{\alpha\:}_{F}$ curves differ somewhat between isometric (broken line) and dynamic (dotted line) contractions at long fascicle lengths (> 70 mm). However, the dotted line is still within the point cloud of isometric $\:{l}_{F}$-$\:{\alpha\:}_{F}$ data.

To demonstrate the validity of $\:{l}_{F}$-$\:{\alpha\:}_{F}$ combinations from isometric measurements for the prediction of dynamic $\:{l}_{F}$-$\:{\alpha\:}_{F}$ combinations, we determined the R^2^ between fitted curves from isometric contractions (Fig. 6, broken lines) and dynamic $\:{l}_{F}$-$\:{\alpha\:}_{F}$ data (Fig. 6, colored dots). The polynomial fits to the isometric data (Fig. 3, broken lines) for the GL and GM achieved similar prediction accuracy for the dynamic data (GL: R^2^ = 0.703, GM: R^2^ = 0.805) as for the isometric data (Fig. 3, GL: R^2^ = 0.800, GM: R^2^ = 0.846). Therefore, the $\:{l}_{F}$-$\:{\alpha\:}_{F}$ curves from the isometric measurements can also be used to predict the dynamic contractions against a sledge load of 125 kg. However, due to the limited number and greater variability of dynamic $\:{l}_{F}$-$\:{\alpha\:}_{F}$ data for SOL (Fig. 6C, green dots), the predictability of dynamic data based on the polynomial fit of isometric data (Fig. 3C) was lower (R^2^ = 0.505).

## Discussion

For GL, GM, and SOL, fascicle length decreased and pennation angle increased with increasing muscle contraction intensity (which corresponds to muscle force in isometric contractions) (Fig. 4). In general, the fascicle length of all muscles decreased with increasing ankle joint angle (from 50° to 120°) and thus decreased muscle length (Fig. 4A, C, E). For the pennation angle, we found muscle specific differences. Although the pennation angles increased with increasing ankle joint angle (from 50 to 120°) for GM and SOL, as well as for GL at muscle contraction intensities > 30%, we found no influence of the ankle joint angle on the GL pennation in the passive condition (0% MVC) or at low forces (up to 20% MVC, Fig. 4B, Supplementary Table S1). This might be partially related to the comparatively low GL pennation angles and the high standard deviation of the measured values, in combination with smaller changes in pennation angle at low muscle contraction intensity.

### $\:{l}_{F}$**-**$\:{\alpha\:}_{F}$ progression

Unfortunately, there are only a few studies in the literature to which we can compare our results on fascicle shortening and changes in pennation angle under similar experimental conditions.

For low contraction intensities (20 − 30% MVC) we found a more pronounced shortening for GM and GL than in the previous study by Heroux et al.^18^. Compared to Narici et al.^19^ we also observed more pronounced GM fascicle shortening (Δ$\:{l}_{F}$ = 28 mm vs. 19 mm) at 110° ankle joint angle for high contraction intensities (90 − 100% MVC). The fascicle shortening ​​values measured in our study (GM: Δ$\:{l}_{F}$ = 32 mm; SOL: Δ$\:{l}_{F}$ = 20 mm) are also larger than the values ​​measured by Maganaris et al.^21^ (GM: Δ$\:{l}_{F}$ = 22 mm; SOL: Δ$\:{l}_{F}$ = 5 mm) and Kawakami et al.^20^ (GM: Δ$\:{l}_{F}$ = 21 mm; SOL: Δ$\:{l}_{F}$ = 12 mm) at 90° ankle angle between rest and 100% MVC. Furthermore, we found larger Δ$\:{l}_{F}$ (26 mm) for GL than Kawakami et al.^20^ reporting Δ$\:{l}_{F}$ = 18 mm. Contrary, we found smaller GL fascicle shortening (Δ$\:{l}_{F}$ = 26 mm) compared to Maganaris et al.^21^ (Δ$\:{l}_{F}$ = 39 mm). However, our relative GL fascicle shortening of 25% is almost similar to Kelp et al.^50^ at 90° ankle joint angle.

For the GM and GL pennation angle we found a more pronounced increase (Fig. 4B, D) than Heroux et al.^18^ for low contraction intensities (20 − 30% MVC). At no point in our study did we observe a decrease in the pennation angle with increasing muscle contraction intensities, as described at the 90° ankle joint angle position for the GL in Heroux et al.^18^. Our measured change in GM pennation angle (Δ$\:{\alpha\:}_{F}$ = 22°) was greater compared with that (Δ$\:{\alpha\:}_{F}$ = 16°) reported by Kawakami et al.^20^. The increase in GM pennation angle between rest and 90% MVC was larger (Δ$\:{\alpha\:}_{F}$ = 28°) in our study than recorded by Narici et al.^19^ at 110° ankle joint angle (Δ$\:{\alpha\:}_{F}$ = 18°). For GM and SOL $\:{\alpha\:}_{F}$ increased almost similar at 90° ankle joint angle during MVC as in Maganaris et al.^21^. For GL, the increase of $\:{\alpha\:}_{F}$ observed in our study was significantly smaller (Δ$\:{\alpha\:}_{F}$ = 8°) than in Maganaris et al.^21^ (Δ$\:{\alpha\:}_{F}$ = 24°). For GL and SOL, the pennation angles observed in our study (GL: Δ$\:{\alpha\:}_{F}$ = 8°; SOL: Δ$\:{\alpha\:}_{F}$ = 17°) are similar to those reported in Kawakami et al.^20^ (GL: Δ$\:{\alpha\:}_{F}$ = 11°; SOL: Δ$\:{\alpha\:}_{F}$ = 19°). Furthermore, we found similar changes in pennation angle at rest for changes in ankle joint angles from 90° to 120° compared to Narici et al.^19^. Moreover, our results largely agree with reported GL, GM and SOL pennation angles at rest for different ankle joint angles^21^.

It is known that SOL, GM and GL show regional differences in muscle architecture^36,37,51^. Differences in fascicle length and pennation angle between our results and the referred studies could therefore be due to the measurement of different regions of the muscles, high variability in muscle architecture between individuals^51^ or age-related differences in the groups of subjects^52^.

Furthermore, there are caveats to the comparison with Kawakami et al.^20^ and Heroux et al.^18^. In the study by Heroux et al.^18^, the subjects performed only a submaximal contraction, with data collected for up to 25% MVC. We compared those points to our data for 20% and 30% MVC, which could have resulted in some differences. Regarding the study by Kawakami et al.^20^ we had to compare our findings for Δ$\:{l}_{F}$ and Δ$\:{\alpha\:}_{F}$ between relaxed and 90% MVC to their reported differences between relaxed and maximal voluntary contraction (100% MVC). Kelp et al.^50^ used a linear regression line for the $\:{l}_{F}\:$-$\:{\:\alpha\:}_{F}$ relation at a 90° ankle joint angle. As they examined one ankle joint angle (90°) only, their regression line corresponds to a limited subsegment of the nonlinear (quadratic fit) $\:{l}_{F}\:$-$\:{\:\alpha\:}_{F}$ relation discovered in our study (Fig. 3A). For all the studies referred to, there are differences in the contraction velocity, which can also influence the results. In our experiments, there was a slow, ramp-like contraction within four seconds, whereas in the compared studies there was no limitation for the contraction velocity.

We observed similar GL fascicle shortening for increasing contraction intensities (0-90% MVC) in isometric contractions at all ankle joint angles (Supplementary Table S1, right column). The observed increase in Δ$\:{\alpha\:}_{F}\:$(from 6° to 15°, Supplementary Table S1, right column) for this given muscle fascicle shortening with increasing contraction intensity could be related to the elasticity of the extracellular matrix and aponeuroses. Higher contraction intensities, corresponding to higher forces, lead to a greater strain of the elastic structures. This could result in a different muscle shape (e.g. larger anatomical cross-section) and a higher pennation angle, despite similar muscle fascicle shortening.

### What does the gearing depend on?

Several studies have shown greater gearing with less muscle force^28–31^. In these studies, contractions are mostly performed with supramaximal muscle stimulation against different isotonic loads. In these investigations, contractions against small loads result in the largest change in the pennation angle and thus the largest G. Since the muscle is activated supramaximally during the shortening in these experiments, the results can only be compared with our isometric data (in which a slow ramp-like muscle contraction intensity occurs within 4 s; Fig. 2) to a limited extent. Looking at an almost fully activated muscle (90% MVC), for changes in ankle joint angle from 50–120°, we observed G values between 1.1 and 1.3 (Fig. 5B, D, F). As expected, lower muscle contraction intensities result in lower Gearing^53^. Consequently, passive changes in ankle joint angle from 50° to 90° resulted in almost no Gearing, especially for GL (G = 1.01 ± 0.03), where no change in pennation angle was found (Fig. 4B). This contradicts the findings by Takahashi et al.^54^, who found a passive gearing in GM, which was even greater (G = 1.4 ± 0.6) than the observed gearing in this study at 90% contraction intensity (Fig. 5D). This could be due to regional differences in the muscle architecture as described by Takahashi et al.^54^ and consequently the investigation of different regions in both studies. Furthermore, Takahashi et al.^54^ recorded the 3D muscle architecture via magnetic resonance imaging whereas we used 2D ultrasound, which simplifies 3D muscle architecture as a projection into a plane. New methods such as 3D ultrasound^55,56^ may in the future provide more detailed insights into muscle gearing during in vivo movements. Interestingly, we found that G depends on muscle length. The greatest G was found for the shortest muscle length (120° ankle joint angle), as muscle fascicles were able to rotate the most (Fig. 4B, D, F) at high ankle joint angles (120°). A comparison with the literature is not possible because, as far as we are aware, there are no other studies on the muscle length dependence of G.

### Isometric and dynamic $\:{l}_{F}$-$\:{\alpha\:}_{F}$ relations

The point clouds for the isometric and dynamic tests are superimposed in Fig. 6. The results for the dynamic contractions for the GL are similar to those found in the study by Randhawa et al.^57^. The polynomial fits to the isometric and dynamic data are almost on top of each other for the GL and GM. With the polynomial fit to the isometric data, good prediction accuracy of the dynamic $\:{l}_{F}$-$\:{\alpha\:}_{F}$ relations (GL: R^2^ = 0.703, GM: R^2^ = 0.805) is achieved. Consequently, at least for the GL and GM, there appears to be a coupling between fascicle length and pennation angle that is independent of contraction mode (isometric or concentric). The volume of the muscle and the muscle fascicles from which it is composed is approximately constant^58,59^. A shortening of the fascicle is thus associated with an increase in the fascicle diameter^60^ and an increase in the pennation angle^61^. Our data suggest that at least for specific muscles (GL and GM) a mechanical coupling exists. A simple 3D architectural model^62^ based on the volume constancy of the muscle supports this suggestion. The model predicts fixed combinations of pennation angles and fascicle lengths depending on the muscle belly length and thus the ankle joint angle. Thus, the mechanical coupling might be caused by the volume constancy of the muscle tissue and muscle specific deformation restrictions. The latter one is affected by the connective tissue surrounding fibers (endomysium), fascicles (perimysium) and whole muscles (epimysium), as well as the aponeuroses serving as the attachment area of muscle fibers. This is important to consider because the properties of these aponeuroses have been shown to depend on their biaxial loading^63^. Furthermore, muscle architecture affects muscle deformation and gearing^30^ and thus may influence $\:{l}_{F}$-$\:{\alpha\:}_{F}$ relations. Muscle gearing appears to depend strongly on the specific muscle and muscle architecture^28^. For example, there are muscles that thicken upon contraction, other muscles show no change in thickness or even a reduction in muscle thickness, which is associated with different muscle gearings^28^. The exact causes of muscle gearing are not yet understood, but gearing might be influenced by the specific muscle architecture, the properties of the extracellular matrix and the transverse forces transmitted to the muscle by the surrounding muscles^64,65^.

## Electronic supplementary material

Below is the link to the electronic supplementary material.

Supplementary Material 1

## Data Availability

The datasets used during the current study are available from the corresponding author on reasonable request.

## Abbreviations

$$\:{\alpha\:}_{F}$$: Pennation angle
G: Muscle gearing
GM: Musculus gastrocnemius medialis
GL: Musculus gastrocnemius lateralis
$\:{l}_{F}$: Muscle fascicle length
MVC: Maximal voluntary contraction
SOL: Musculus soleus
