# Supplementary material for: Impact of contraction intensity and ankle joint angle on calf muscle fascicle length and pennation angle during isometric and dynamic contractions
Source: Sci Rep. 2024 Oct 22;14:24929. doi: 10.1038/s41598-024-75795-2 (PMC11496514; doi:10.1038/s41598-024-75795-2)
Supplement: Supplementary file 1 — Supplementary Material 1 [file 41598_2024_75795_MOESM1_ESM.pdf]

Supplementary Information for the manuscript:

Impact of contraction intensity and ankle joint angle on calf muscle fascicle length and pennation angle during isometric and dynamic contractions

Corinna Coenning, Volker Rieg, Tobias Siebert, and Veit Wank

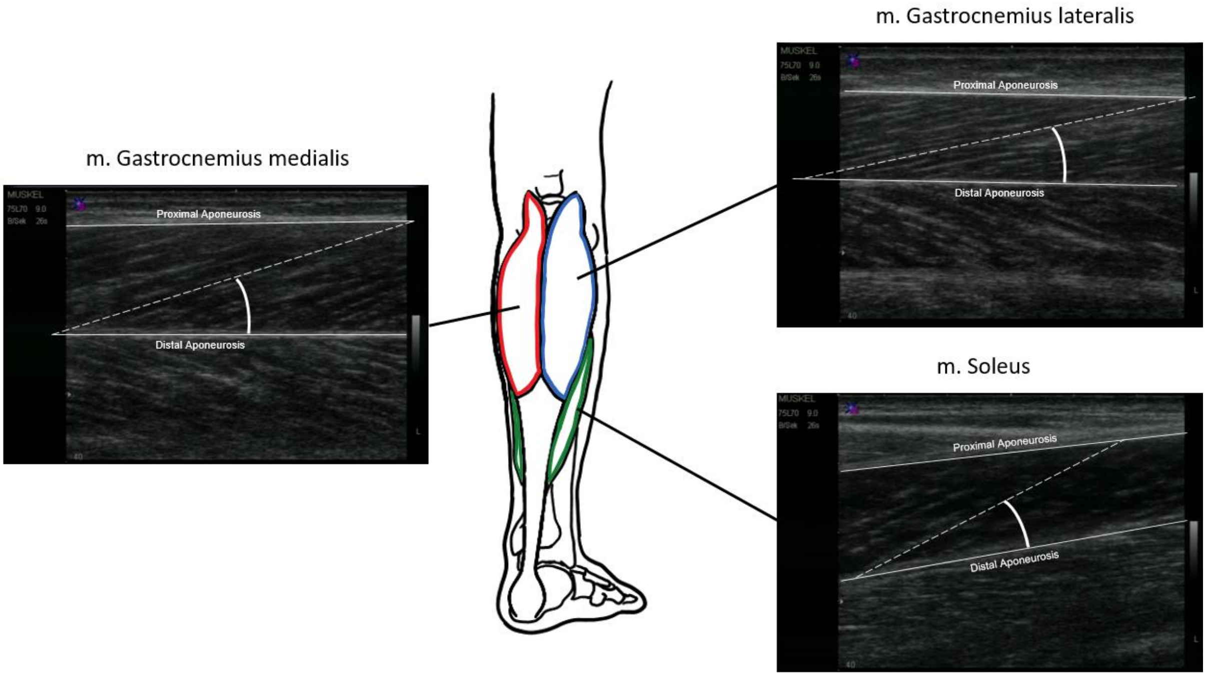

Supplementary Fig. S1. The Anatomy of the m. Triceps surae consisting of GL, GM, and SOL. Each considered muscle is marked in a different color: GL (blue), GM (red), SOL (green). For each muscle an ultrasound image at 90° ankle joint angle at rest is shown. For each image the two solid lines marked the proximal (upper) and distal (lower) aponeurosis, the dashed line marked an example muscle fascicle, the curved line visualized the pennation angle. SOL, GM and GL exhibit complex muscle architectures with regional variations. GM fascicles arose from the distal aponeurosis with variable lengths and angles both in sagittal and coronal planes (Takahashi et al. 2022). GL shows substantial variability in fascicle lengths between different muscle regions (Aeles et al. (2022). The SOL consists of four compartments, which exhibit homogeneity in fascicle lengths and inhomogeneity in pennation angles (Bolsterlee et al. 2018). We examined the unipennate posterior portion of SOL. For GM and GL we examined the part with the largest CSA.

Supplementary Table S1. M. gastrocnemius lateralis (GL) mean l_F and α_F depending on ankle joint angle and muscle force. * p<0.05

**GL**			**Muscle Force [%MVC]**										
			**0% passive**	**10%**	**20%**	**30%**	**40%**	**50%**	**60%**	**70%**	**80%**	**90%**	**Δl_F and $\Delta \alpha_F$ (0% - 90% contraction intensity)**
**Ankle Joint Angle [°]**	**50°**	l_F [mm]	88±14	84±16	78±16	76±16	72±15	69±15	67±14	66±14	65±15	60±15	**27***
		α_F [°]	11±2	12±3	12±3	13±3	13±3	14±3	15±3	15±3	15±3	17±3	**6***
	**60°**	l_F [mm]	81±15	78±18	75±17	72±16	69±14	67±15	64±13	63±15	61±14	58±13	**24***
		α_F [°]	11±2	12±2	12±3	13±3	13±2	14±2	15±2	15±3	16±3	17±3	**6***
	**70°**	l_F [mm]	85±13	80±14	77±14	73±13	70±12	68±12	65±11	62±11	60±11	58±10	**27***
		α_F [°]	11±2	12±2	12±2	13±2	13±2	14±2	14±2	15±2	16±3	17±3	**6***
	**80°**	l_F [mm]	79±9	75±12	71±12	68±12	65±10	62±11	59±11	56±10	55±9	53±8	**26***
		α_F [°]	11±1	17±1	12±1	13±1	14±2	14±2	15±3	16±3	17±3	18±3	**7***
	**90°**	l_F [mm]	77±16	72±16	68±14	65±12	62±12	60±11	58±12	56±12	54±11	52±10	**26***
		α_F [°]	12±3	12±3	13±3	13±3	14±3	15±3	16±3	17±3	18±3	20±3	**8***
	**100°**	l_F [mm]	74±14	72±18	70±19	67±16	62±10	58±7	54±7	52±8	50±8	45±6	**29***
		α_F [°]	12±2	12±3	12±3	13±3	14±3	16±2	18±3	19±3	21±4	23±4	**11***
	**110°**	l_F [mm]	73±13	69±12	64±13	64±.13	57±11	54±9	52±10	50±10	47±9	44±8	**28***
		α_F [°]	12±3	13±3	13±4	14±4	16.±4	18±4	19±4	21±4	22±5	23±5	**12***
	**120°**	l_F [mm]	71±13	69±13	66±12	62±12	59±10	54±9	52±9	49±9	48±9	42±8	**29***
		α_F [°]	12±3	13±3	14±3	14±4	17±3	19±3	21±3	22±3	24±5	27±6	**15***
	**50°-120°**	Δl_F [mm]	**17***	**15***	**12***	**14***	**13***	**15***	**15***	**17***	**17***	**18***	
	**50°-120°**	$\Delta \alpha_F$ [°]	**1**	**1**	**2**	**2**	**4***	**5***	**6***	**7***	**9***	**10***	

Supplementary Table S2. M. gastrocnemius medialis (GM) mean l_F and α_F depending on ankle joint angle and muscle force. * p<0.05

**GM**	**Muscle Force [%MVC]**										
	**0% passive**	**10%**	**20%**	**30%**	**40%**	**50%**	**60%**	**70%**	**80%**	**90%**	**Δl_F and $\Delta \alpha_F$ (0% - 90% contraction intensity)**

Ankle joint angle [°]	50°	l_F [mm]	76±10	71±8	68±8	65±7	63±7	60±7	56±6	55±7	54±7	52±7	**24***
		α_F [°]	15±1	16±2	17±2	18±2	19±2	19±2	21±2	21±2	22±2	22±2	**7***
	60°	l_F [mm]	71±8	66±9	63±9	61±8	58±8	56±7	53±6	50±5	48±4	44±2	**27***
		α_F [°]	15±2	16±2	17±3	18±3	19±3	20±3	21±3	22±3	23±3	24±3	**9***
	70°	l_F [mm]	64±4	62±4	60±4	57±5	54±5	52±6	48±8	45±8	42±9	41±8	**23***
		α_F [°]	16±1	17±1	17±1	18±1	19±1	21±2	22±3	24±4	27±5	27±6	**11***
	80°	l_F [mm]	63±9	60±8	58±8	56±8	52±8	48±8	45±8	42±8	39±8	37±5	**26***
		α_F [°]	17±3	18±3	19±3	20±4	21±4	24±5	25±4	27±5	29±6	31±5	**14***
	90°	l_F [mm]	62±10	58±8	55±8	51±10	48±8	43±8	41±8	38±8	33±6	30±5	**32***
		α_F [°]	17±2	18±2	19±2	22±3	23±3	26±4	28±5	30±5	35±4	39±5	**22***
	100°	l_F [mm]	54±6	52±6	47±6	43±7	39±5	37±5	34±4	32±4	30±4	28±3	**26***
		α_F [°]	18±2	19±2	23±4	26±4	28±4	31±5	34±5	36±5	39±4	42±5	**24***
	110°	l_F [mm]	53±10	49±7	44±7	39±7	34±6	31±5	28±3	27±3	26±3	25±2	**28***
		α_F [°]	20±3	22±4	25±4	29±6	34±6	38±6	42±6	44±6	46±6	48±5	**28***
	120°	l_F [mm]	47±7	42±7	38±5	35±4	32±5	30±	28±4	27±3	26±3	25±3	**22***
		α_F [°]	21±2	25±3	29±3	32±4	36±5	38±5	42±5	44±4	46±5	55±4	**24***
	50°-120°	Δ l_F [mm]	**28***	**28***	**30***	**30***	**31***	**30***	**28***	**28***	**28***	**27***	
	50°-120°	Δ α_F [°]	**6***	**9***	**13***	**14***	**17***	**19***	**21***	**23***	**25***	**33***	

Supplementary Table S3. M. soleus (SOL) mean l_F and α_F depending on ankle joint angle and muscle force. * p<0.05

SOL			Muscle Force [%MVC]										
			**0% passive**	**10%**	**20%**	**30%**	**40%**	**50%**	**60%**	**70%**	**80%**	**90%**	**Δl_F and Δ α_F (0% - 90% contraction intensity)**
**Ankle joint angle [°]**	**50°**	l_F [mm]	67±10	62±9	58±9	55±10	53±10	51±10	49±11	48±10	47±11	41±10	**26***
		α_F [°]	14±3	15±3	16±3	17±4	18±4	19±4	20±5	20±5	21±5	24±5	**11***
	**60°**	l_F [mm]	62±10	58±10	54±9	54±9	48±9	46±8	44±8	41±8	39±6	38±6	**24***
		α_F [°]	15±2	16±2	17±2	16±3	19±3	20±3	22±4	23±4	24±5	25±6	**10***
	**70°**	l_F [mm]	61±8	57±8	56±7	50±7	50±7	46±7	44±7	41±7	40±7	38±8	**23***
		α_F [°]	15±3	15±3	16±3	18±3	18±3	20±3	21±4	23±4	24±5	25±5	**10***
	**80°**	l_F [mm]	55±5	52±4	49±4	46±4	43±5	42±5	40±5	38±5	36±4	33±5	**22***
		α_F [°]	16±2	17±2	18±2	19±3	21±3	22±3	23±3	25±3	26±4	29±5	**13***
	**90°**	l_F [mm]	48±7	45±6	41±4	38±3	36±3	34±3	32±2	31±3	29±2	27±2	**20***
		α_F [°]	20±4	22±4	24±3	25±4	27±4	29±4	31±3	32±4	34±4	37±3	**17***
	**100°**	l_F [mm]	45±6	44±5	41±4	40±3	37±3	35±3	34±3	33±3	32±3	27±2	**19***

		α_F [°]	21±6	22±6	24±6	27±5	29±5	31±4	33±5	34±5	35±6	40±4	**19***
	**110°**	l_F [mm]	40±4	38±6	35±4	36±4	31±3	30±2	29±2	27±2	26±2	26±1	**13***
		α_F [°]	24±5	26±4	28±3	30±3	32±3	34±4	36±4	38±5	39±5	40±4	**16***
	**120°**	l_F [mm]	43±13	40±13	38±11	33±13	35±12	33±11	33±11	32±11	27±12	24±3	**18***
		α_F [°]	25±8	27±8	29±8	31±9	32±10	34±10	35±11	36±11	41±11	45±9	**20***
	**50°-120°**	$\Delta\, l_F$ [mm]	**24***	**22***	**20***	**22***	**18***	**18***	**16***	**16***	**20***	**17***	
	**50°-120**	$\Delta\, \alpha_F$ [°]	**11***	**12***	**13***	**14***	**14***	**15***	**15***	**16***	**20***	**21***	

Supplementary Note

Fascicle length decreased and pennation angle increased in GM and SOL with increasing muscle contraction intensity (Fig. 4C-F). Thus, for all muscles (GL, GM, SOL), the fascicle length decreased with increasing muscle contraction intensity. The pennation angle increased with increasing ankle joint angle (from 50° to 120°), except for GL, which showed no change in pennation angle for low muscle contraction intensities (0% to 20% MVC, Fig. 4B, Supplementary Table S1).

Considering the entire possible measurement range, i.e., starting from the passive condition at 50° up to 90% MVC at 120° ankle joint angle, we found for all muscles (GL, GM, SOL) fascicle shortening by about half and an approximately threefold increase in the pennation angle. The mean muscle fascicle shortening of GM (≈51 mm, from 76±10 mm to 25±3 mm, significant with $p<0.01$) and GL (≈ 46 mm, from 88±14 mm to 42±8 mm, significant with $p<0.01$) were almost identical (Fig. 4A, C). However, mean changes in pennation angle were greater in GM (≈ 40°, from 15±1° to 55±4°, significant at $p<0.01$; Fig. 4D) than in the GL (≈16°, from 11±2° to 27±6°, significant at $p<0.01$; Fig. 4B). Due to the shorter muscle fascicles, SOL exhibited the smallest change in length (≈43 mm, from 67±10 mm to 24±3 mm; $p<0.01$, Fig. 4E). The corresponding changes in SOL pennation angle (≈ 31°, from 14±3° to 45±9°, significant with $p<0.01$; Fig. 4F) were similar to those in the GM (≈ 40°, from 15±1° to 55±4°, significant with $p<0.01$; Fig. 4D).
